# The association between the non-HDL-cholesterol to HDL-cholesterol ratio and 28-day mortality in sepsis patients: a cohort study

**DOI:** 10.1038/s41598-022-07459-y

**Published:** 2022-03-03

**Authors:** Le Chang, Xinglin Chen, Cheng Lian

**Affiliations:** 1 Department of Orthopedics, Xijing Hospital, The Fourth Military Medical University, Changle West Road No. 127, Xi’an, 710032 Shaanxi China; 2 Department of Geriatrics, Union Hospital, Tongji Medical College, Huazhong University of Science and Technology, Wuhan, China; 3 Department of Epidemiology and Biostatistics, Empower U, X&Y Solutions Inc., Boston, USA; 4 Department of Cardiology, Xi’an International Medical Center Hospital, Xitai Road No. 777, Xi’an, 710032 Shaanxi China

**Keywords:** Risk factors, Infectious diseases

## Abstract

The focus of this study was to explore the association between the non-HDL-cholesterol to HDL-cholesterol (non-HDLc/HDLc) ratio and mortality in septic patients. This was a retrospective cohort study of patients with sepsis in the eICU Collaborative Research Database (eICU-CRD) from 208 distinct ICUs across the United States between 2014 and 2015 that explored. All-cause mortality within 28 days after ICU admission. A multivariable logistic regression model was used to estimate the risk of death. Of the 724 patients with a median age of 68 years, 43 (5.94%) died within 28 days after ICU admission. When the non-HDLc/HDLc ratio was < 3.3, the mortality rate decreased with an adjusted odds ratio (OR) of 0.60 (95% CI 0.37–0.99, *P* = 0.043) for every 1 increment in the non-HDLc/HDLc ratio. When the non-HDLc/HDLc ratio was ≥ 3.3, the mortality rate increased with an adjusted OR of 1.28 (95% CI 1.01–1.62, *P* = 0.039) for every one increment in the non-HDLc/HDLc ratio. For patients with sepsis, the association between the non-HDLc/HDLc ratio and the 28-day mortality risk was a U-shaped curve. A higher or lower non-HDLc/HDLc ratio was associated with an increased risk of 28-day mortality.

## Introduction

Sepsis is a common and lethal syndrome. The systemic inflammatory response is biologically complex, redundant, and activated by infectious and noninfectious triggers. Its manipulation can bring benefits and harm^1^. Therefore, a closer examination of the phenotypes and subphenotypes of patients who exhibit strong survival signals in sepsis may enable us to understand novel mechanisms for improving treatment.

High-density lipoprotein-cholesterol (HDLc) or low-density lipoprotein-cholesterol (LDLc) cholesterol is inversely associated with coronary heart disease (CHD)^2,3^ and can respond differently to changes in diet and treatment. A pool of 458 population-based studies involving 82.1 million participants in 23 countries in Asia and in western Korea found that HDL cholesterol increased in many Western countries, Japan and South Korea^4^. The non-HDLc to high-HDLc (non-HDLc/HDLc) ratio can be obtained from the standard lipid profile without additional cost and is highly correlated with levels of LDL particle number^4^, which has been confirmed as a strong cardiovascular risk marker by multiple studies^5,6^. We previously showed that non-HDLc/HDLc ratio is an independent risk factor for the development of chronic kidney disease (CKD)^7^.

A recent clinical study showed that during a median follow-up of 1.72 years, the mortality rate associated with the range of LDLc/HDLc ratios was U-shaped in hypertensive patients^8^. A retrospective cohort study conducted at a tertiary-care academic medical centre found an association of presepsis baseline LDLc and triglyceride levels with hospital mortality in sepsis. This showed that high (≥ 200 mg/dL) or low levels (≤ 75 mg/dL) of both led to increased mortality. However, there is a lack of evidence to guide the emergency management of patients with sepsis.

We hypothesised that in patients with sepsis, even high and low non-HDLc/HDLc ratios are associated with a higher risk of all-cause mortality within 28-days after admission to the intensive care unit (ICU). In this retrospective multicentre cohort study, we used the eICU Collaborative Research Database (eICU-CRD) from the Philips Healthcare eICU program from 208 distinct ICUs in the United States between 2014 and 2015. We aimed to explore the threshold of the non-HDLc/HDLc ratio where the risk of death significantly increases, which is a high priority in patients with sepsis.

## Methods

### Data source

This was a retrospective observational study in which data were extracted from an online international database, the eICU Collaborative Research Database (eICU-CRD)^9^. The eICU-CRD is a multicentre ICU database with high granularity data for over 200,000 admissions to ICUs monitored by eICU programme across the United States. From 2014 to 2015, all data were automatically stored through the Philips Healthcare eICU program and retrieved electronically^9^. The eICU-CRD has been used for observational research^10–12^. It is possible to access this database by passing an examination and obtaining a certification in accordance with the data usage agreement of the PhysioNet Review Board. The utilized database is released under the Health Insurance Portability and Accountability Act (HIPAA) Safe Harbor provision. Access to the data was approved after completing the Collaborative Institutional Training Initiative (CITI) program “Data or Specimens Only Research”. The study was exempt from approval from the institutional review board of the Massachusetts Institute of Technology (our record ID: 40859994) due to the retrospective design, lack of direct patient intervention, and the security schema for which the reidentification risk was certified as meeting safe harbor standards by Privacert (Cambridge, MA). Informed consent was waived for the same reason. The study was performed in accordance with the Declaration of Helsinki. All methods were performed in accordance with the relevant guidelines and regulations.

### Study population

**Figure 1:**
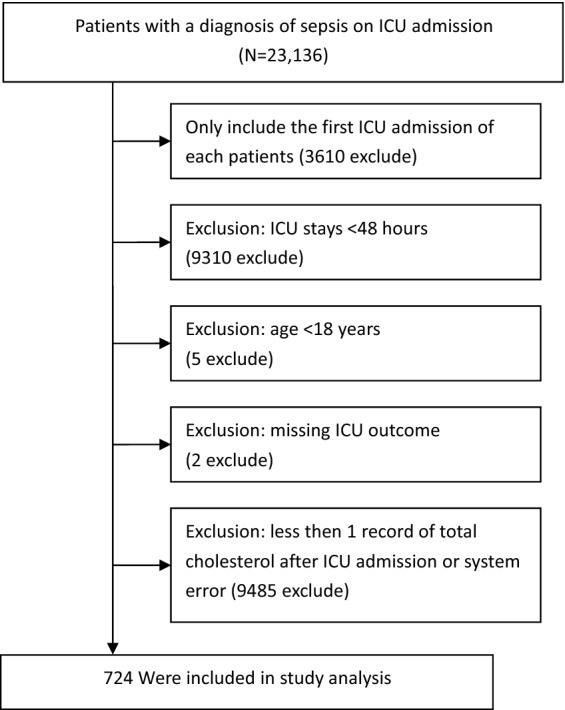
Flow chart of study population. *ICU* intensive care unit.

All patients diagnosed with sepsis on admission to the ICU were included. Sepsis was defined as suspected or documented infection plus an acute increase in Sequential Organ Failure Assessment (SOFA) score greater than 2 points^13^ recorded in the Acute Physiology and Chronic Health Evaluation (APACHE) IV dataset^14^. Infection was identified from the ICD-9 code in the eICU Collaborative Research Database. The following exclusion criteria were used: (1) not first ICU admission, (2) ICU stay < 48 h, (3) < 18 years old, (4) missing ICU outcome, and (5) missing total cholesterol after ICU admission or system error. The study flowchart was presented in Fig. 1.

### Variables

The eICU database includes demographic records, physiological indicators of bedside monitors, diagnosis via the International Classification of Diseases, 9th Edition, Clinical Modification (ICD-9-CM) codes, and other laboratory data obtained during routine medical care.

All participant data within the first 24 h after hospital admission were collected from the eICU-CRD. The physiological variables, including temperature (°C), respiratory rate, heart rate (HR) and mean arterial pressure (MAP), were obtained from the apacheApsVar table. Baseline characteristics such as age, gender, ethnicity, and weight were collected from the tables of patient and apachePatientResult tables. The laboratory indices of lactate level, total cholesterol, triglycerides, HDLc, and LDLc were collected from the laboratory tables. Non-HDLc refers to total cholesterol (TC), including cholesterol in atherogenic lipoproteins, minus HDLc. Comorbidities including acute immunodeficiency syndrome (AIDS), hepatic failure, metastatic cancer, leukaemia, immunosuppression, and cirrhosis were extracted from the APACHE IV score. Septic shock was defined as sepsis with a septic shock diagnosis code and was extracted from the table diagnosis table. Severity at admission was measured by the SOFA score, Apache IV score, Glasgow Coma Scale (GCS) score, and Acute Physiology Score III.

### Outcomes

The outcome of the study was all-cause ICU mortality within 28 days after admission to the ICU. In the supplemental analysis, we also analysed the 14-day mortality rate after admission to the ICU.

### Statistical analysis

Continuous variables are described as the means ± SD or median and interquartile ranges (IQR). Categorical data are presented as numbers and percentages. The difference according to the tertiles of the non-HDLc/HDLc ratio was compared using one-way analysis of variance (ANOVA) for continuous data and chi-squared tests for categorical variables.

We used a generalized additive model (GAM) to investigate the dose–response relationship between the non-HDLc/HDLc ratio and mortality (Fig. 1). We used a logistic regression model to estimate the association between the non-HDLc/HDLc ratio and 28-day mortality. The results are presented as odds ratios (ORs) with its 95% confidence intervals (95% CIs). Crude regression estimates are presented and estimates adjusted for covariates are presented. We selected these confounders on the basis of their association with the outcomes of interest or changes in effect estimates of more than 10%^15^. After considering the clinical significance, we adjusted for the following covariates: age (years), sex, weight, heart rate, lactate level, Apache IV score, SOFA score, septic shock, and site of infection.

We then used a two-piece-wise linear regression model to examine the threshold effect of the non-HDLc/HDLc ratio on mortality (Table 3). The turning point for the non-HDLc/HDLc ratio was determined using “exploratory” analyses, which is to move the trial turning point along the pre-defined interval and pick up the one which gave maximum model likelihood. We also performed a log-likelihood ratio test and compared the one-line linear regression model with the two-piece-wise linear model. We used the bootstrap resampling method to calculate the 95% CI for the turning point^16^, as described in the previous analysis^17,18^.

To examine the robustness of the results, we conducted sensitivity analyses. Dummy variables were used to indicate missing covariate values, which was performed when continuous variables were missing more than 1% of value. We explored the potential for unmeasured confounding between the non-HDLc/HDLc ratio and 28-day mortality by calculating E-values^19^. The E-value quantifies the required magnitude of an unmeasured confounder that could negate the observed association between the non-HDLc/HDLc ratio and 28-day mortality. The two-sided alpha level was set at 0.05. All the statistical analyses were performed using the EmpowerStats (www.empowerstats.com, X&Y solutions, Inc. Boston MA) and R software version 3.6.1 (http://www.r-project.org).

### Ethics approval and consent to participate

Data was extracted from the eICU Collaborative Research Database (eICU-CRD)^9^ in accordance with the data usage agreement (our record ID: 40859994) by the PhysioNet review committee. The utilized database is released under the Health Insurance Portability and Accountability Act (HIPAA) safe harbor provision. This was a retrospective analysis based on an anonymous database for researchers and did not require ethical approval from the local ethics committee.

## Results

### Baseline characteristics

Data from 724 patients were analysed. The median age was 68 years (IQR 58–78 years). 334 patients (46.1%) were women. Table 1 compares the patient's demographics, vital signs, laboratory results, site of infection, and severity of illness through the tertiles of the non-HDLc/HDLc ratio. Compared with subjects in the lowest tertile of the non-HDLc/HDLc ratio, subjects in the highest tertile were younger and had higher weight at admission.

**Table 1:** Baseline characteristics and 28-day mortality according to the tertiles of the non-HDLc/HDLc ratio (n = 724).

Parameters	Non-HDLc/HDLc ratio			*P* value
	Tertile 1 0.28–2.24 n = 241	Tertile 2 2.25–3.83 n = 241	Tertile 3 3.84–10 n = 242	
**Demographics**				
Age (year)	69.6 ± 14.0	67.2 ± 14.7	63.3 ± 15.9	< 0.001
Sex				
Male	124 (51.5%)	130 (53.9%)	136 (56.2%)	0.578
Female	117 (48.5%)	111 (46.1%)	106 (43.8%)	
Ethnicity				
Caucasian	173 (71.8%)	168 (69.7%)	177 (73.1%)	0.904
African American	29 (12.0%)	35 (14.5%)	26 (10.7%)	
Hispanic	13 (5.4%)	14 (5.8%)	15 (6.2%)	
Asian	3 (1.2%)	3 (1.2%)	5 (2.1%)	
Native American	1 (0.4%)	2 (0.8%)	0 (0%)	
Other/unknown	22 (9.1%)	19 (7.9%)	19 (7.9%)	
Admission weight (kg)	80.4 ± 24.2	85.2 ± 28.2	91.0 ± 30.8	< 0.001
Period				
2014	104 (43.2%)	121 (50.2%)	110 (45.5%)	0.285
2015	137 (56.8%)	120 (49.8%)	132 (54.5%)	
**Vital signs**				
Temperature (°C)	36.6 ± 1.1	36.7 ± 1.2	36.6 ± 1.3	0.759
Respiratory rate (bpm)	30.4 ± 14.6	30.1 ± 14.8	30.5 ± 14.4	0.936
Heart rate (/min)	109.5 ± 29.1	111.1 ± 28.7	115.7 ± 25.6	0.043
MAP (mmHg)	79.7 ± 44.9	87.3 ± 47.6	77.2 ± 41.8	0.038
**Laboratory data**				
Lactate level (mmol/L)	1.9 (1.2–2.9)	1.7 (1.3–2.7)	2.0 (1.3–3.1)	0.555
Total cholesterol (mg/dL)	105.5 ± 35.7	118.6 ± 41.7	123.0 ± 50.8	< 0.001
Triglycerides (mg/dL)	79 (60–107)	112 (79–158)	154 (121–216)	< 0.001
HDLc (mg/dL)	42.6 ± 14.8	30.0 ± 11.2	19.2 ± 9.2	< 0.001
LDLc (mg/dL)	46.0 ± 23.5	64.1 ± 29.6	70.2 ± 39.7	< 0.001
non-HDLc/HDL ratio	1.6 (1.2–1.9)	3.0 (2.6–3.3)	5.4 (4.5–6.6)	< 0.001
Septic shock	129 (53.5%)	142 (58.9%)	151 (62.4%)	0.138
**Site of infection**				
Sepsis, pulmonary	112 (46.5%)	101 (41.9%)	99 (40.9%)	< 0.001
Sepsis, renal/UTI (including bladder)	55 (22.8%)	46 (19.1%)	62 (25.6%)	
Sepsis, GI	26 (10.8%)	21 (8.7%)	24 (9.9%)	
Sepsis, unknown	22 (9.1%)	29 (12.0%)	22 (9.1%)	
Sepsis, cutaneous/soft tissue	18 (7.5%)	25 (10.4%)	15 (6.2%)	
Sepsis, other	8 (3.3%)	19 (7.9%)	20 (8.3%)	
**Severity of illness**				
SOFA score	3.0 (1.0–5.0)	3.0 (1.0–4.0)	3.0 (1.0–5.0)	0.626
GCS score	11.8 ± 3.7	11.8 ± 3.8	12.2 ± 3.6	0.398
Apache IV score	73.3 ± 22.4	73.6 ± 24.8	72.7 ± 24.5	0.920
Acute Physiology Score III	58.3 ± 22.8	60.1 ± 23.8	60.8 ± 22.9	0.523
**Comorbidities**				
AIDS	0 (0.0%)	0 (0.0%)	1 (0.4%)	0.370
Hepatic failure	4 (1.7%)	2 (0.8%)	3 (1.2%)	0.710
Leukaemia	1 (0.4%)	4 (1.7%)	1 (0.4%)	0.338
Metastatic cancer	3 (1.3%)	3 (1.2%)	4 (1.7%)	0.908
Immunosuppression	8 (3.3%)	4 (1.7%)	6 (2.5%)	0.500
Cirrhosis	5 (2.1%)	3 (1.2%)	3 (1.2%)	0.685
**28-day mortality**				
No	224 (92.9%)	233 (96.7%)	224 (92.6%)	0.107
Yes	17 (7.1%)	8 (3.3%)	18 (7.4%)	

Data are expressed as the mean ± SD, median (interquartile range), or percentage.

Among the 724 patients, the amount of missing values for the covariates were 11 (1.5%) for admission weight, 45 (6.2%) for temperature, 9 (1.2%) for respiratory rate, 5 (0.7%) for heart rate, 5 (0.7%) for MAP, 205 (28.3%) for lactate level, 78 (10.8%) for Apache IV score, 78 (10.8%) for Acute Physiology Score III, 2 (0.3%) for triglycerides, 178 (24.6%) for LDLc, 7 (1.0%) for SOFA score, and 16 (2.2%) for GCS score.

*BP* blood pressure, *GCS* Glasgow Coma Scale, *HDLc* high-density lipoprotein cholesterol, *LDLc* low-density lipoprotein cholesterol, *MAP* mean arterial pressure, *SOFA* Sequential Organ Failure Assessment, *UTI* urinary tract infection.

### 28-day mortality

The 28-day ICU mortality rate was 43/724 = 5.94% (95% CI 4.22–7.66) in our cohort. The 28-day mortality rate from the lowest tertile (0.28–2.24) to the highest (3.84–10) non-HDLc/HDLc ratio was 17 (7.1%), 8 (3.3%), and 18 (7.4%) (Table 1).

### Unadjusted association between baseline variables and 28-day mortality

Table 2 shows the univariate logistic models. The analysis showed that compared with the low temperature group (30–36.38 °C), the high temperature group (36.4–36.72 °C) had a lower risk of mortality (OR = 0.28, 95% CI 0.11–0.72, *P* = 0.0082). Compared with the low SOFA score group (all = 0), the middle and high groups (range 1–3 and 4–15) had a higher risk of mortality (OR = 9.28, 95% CI 1.21–70.97, *P* = 0.0318; OR = 14.61, 95% CI 1.97–108.56, *P* = 0.0088).

**Table 2:** The unadjusted association between baseline variables and 28-day mortality (n = 724).

Exposure	Statistics	Odds ratio (95% CI)	*P* value
Non-HDLc/HDL ratio	3.43 ± 2.01	1.10 (0.96, 1.27)	0.1682
**Non-HDLc/HDL ratio tertile**			
Low	241 (33.29%)	Reference	
Middle	241 (33.29%)	0.45 (0.19, 1.07)	0.0707
High	242 (33.43%)	1.06 (0.53, 2.11)	0.8707
**Age (years) tertile**			
18–61	240 (33.15%)	Reference	
62–74	238 (32.87%)	1.88 (0.85, 4.17)	0.1189
75–89	246 (33.98%)	1.49 (0.66, 3.39)	0.3380
**Septic shock**			
No	302 (41.71%)	Reference	
Yes	422 (58.29%)	1.10 (0.59, 2.07)	0.7653
**Source of infection**			
Sepsis, pulmonary	312 (43.09%)	Reference	
Sepsis, renal/UTI (including bladder)	163 (22.51%)	0.56 (0.24, 1.34)	0.1958
Sepsis, GI	71 (9.81%)	1.16 (0.45, 2.96)	0.7566
Sepsis, unknown	73 (10.08%)	1.13 (0.44, 2.87)	0.8050
Sepsis, cutaneous/soft tissue	58 (8.01%)	0.22 (0.03, 1.67)	0.1428
Sepsis, other	47 (6.49%)	^a^	0.9866
**Gender**			
Male	390 (53.87%)	Reference	
Female	334 (46.13%)	0.54 (0.28, 1.05)	0.0691
**Ethnicity**			
Caucasian	518 (71.55%)	Reference	
African American	90 (12.43%)	0.73 (0.25, 2.12)	0.5641
Hispanic	42 (5.80%)	0.79 (0.18, 3.40)	0.7468
Asian	11 (1.52%)	1.57 (0.19, 12.67)	0.6715
Native American	3 (0.41%)	^a^	0.9878
Other/Unknown	60 (8.29%)	1.43 (0.53, 3.82)	0.4782
**Hospital discharge year**			
2014	335 (46.27%)	Reference	
2015	389 (53.73%)	0.99 (0.53, 1.84)	0.9739
**Admission weight, kg Tertile**			
27.21–70.2	237 (33.24%)	Reference	
70.3–90.9	235 (32.96%)	1.15 (0.56, 2.37)	0.6960
91–227	241 (33.80%)	0.71 (0.32, 1.57)	0.3969
**Temperature (°C) Tertile**			
30–36.38	220 (32.40%)	Reference	
36.4–36.72	217 (31.96%)	0.28 (0.11, 0.72)	0.0082
36.8–41.8	242 (35.64%)	0.71 (0.36, 1.40)	0.3226
**Respiratory rate (bpm) Tertile**			
4–27	227 (31.75%)	Reference	
28–36	248 (34.69%)	1.50 (0.66, 3.37)	0.3301
37–60	240 (33.57%)	1.65 (0.74, 3.69)	0.2193
**Heart rate (bpm) Tertile**			
26–103	224 (31.15%)	Reference	
104–122	245 (34.08%)	1.20 (0.52, 2.79)	0.6736
123–201	250 (34.77%)	1.86 (0.85, 4.07)	0.1193
**MAP (mmHg) Tertile**			
40–51	238 (33.10%)	Reference	
52–71	240 (33.38%)	1.13 (0.55, 2.33)	0.7328
72–199	241 (33.52%)	0.71 (0.32, 1.58)	0.4031
**Lactate level (mmol/L) Tertile**			
0.40–1.40	171 (32.95%)	Reference	
1.50–2.40	171 (32.95%)	0.57 (0.22, 1.47)	0.2432
2.50–16.55	177 (34.10%)	1.69 (0.80, 3.57)	0.1707
**SOFA score Tertile**			
0–0	152 (21.20%)	Reference	
1–3	259 (36.12%)	9.28 (1.21, 70.97)	0.0318
4–15	306 (42.68%)	14.61 (1.97, 108.56)	0.0088
**GCS score group**			
3–10	216 (30.51%)	Reference	
11–14	207 (29.24%)	0.62 (0.28, 1.34)	0.2229
15–15	285 (40.25%)	0.53 (0.25, 1.10)	0.0871
**APACHE IV score Tertile**			
13–60	212 (32.82%)	Reference	
61–81	217 (33.59%)	3.31 (1.06, 10.33)	0.0389
82–164	217 (33.59%)	5.57 (1.88, 16.52)	0.0020
**Acute Physiology Score III Tertile**			
4–47	213 (32.97%)	Reference	
48–67	214 (33.13%)	1.83 (0.60, 5.54)	0.2876
68–147	219 (33.90%)	5.12 (1.92, 13.68)	0.0011

Data are expressed as the mean ± SD, or percentage.

*BP* blood pressure, *GCS* Glasgow Coma Scale, *HDLc* High-density lipoprotein cholesterol, *LDLc* Low-density lipoprotein cholesterol, *MAP* Mean Arterial Pressure, *SOFA* Sequential Organ Failure Assessment, *UTI* Urinary Tract Infection.

^a^The model failed because of the small sample size.

### Identification of nonlinear relationship

**Figure 2:**
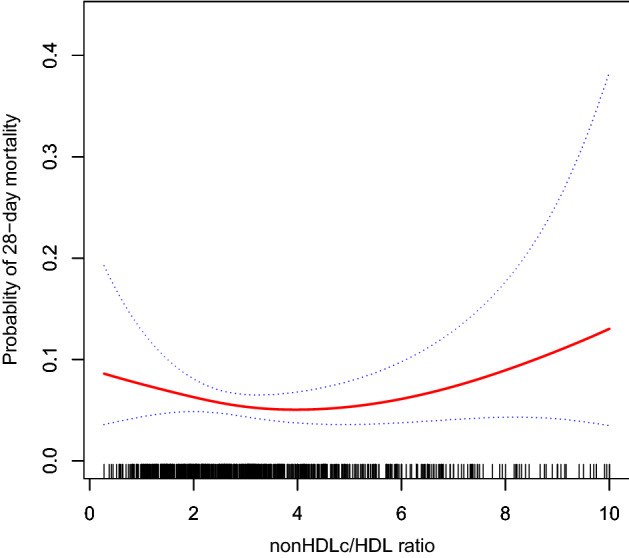
Associations between the non-HDLc/HDLc ratio and 28-day mortality in all patients with sepsis. A threshold, nonlinear association between the non-HDLc/HDLc ratio and 28-day mortality was found in a generalized additive model (GAM). Solid rad line represents the smooth curve fit between variables. Blue bands represent the 95% of confidence interval from the fit. Adjusted for age (years), sex, weight, heart rate, lactate level, Apache IV score, SOFA score, septic shock and site of infection.

We observed a nonlinear dose–response relationship between the non-HDLc/HDLc ratio and mortality (Fig. 2 and Table 3). When the non-HDLc/HDLc ratio was < 3.3, the mortality rate decreased with an adjusted OR of 0.60 (95% CI 0.37–0.99, *P* = 0.043) for every 1 increment in the non-HDLc/HDLc ratio. When the non-HDLc/HDLc ratio was ≥ 3.3, the mortality rate increased with an adjusted OR of 1.28 (95% CI 1.01–1.62, *P* = 0.039) for every 1 increment in the non-HDLc/HDLc ratio. As the non-HDLc/HDLc ratio increased per SD, when the non-HDLc/HDLc ratio was ≥ 3.3, the OR for mortality was 1.64 (95% CI 1.02–2.63, *P* = 0.039). When the non-HDLc/HDLc ratio was < 3.3, the OR for mortality was 0.36 (95% CI 0.13–0.97, *P* = 0.043) (Table 3).

**Table 3:** Threshold effect analysis of the non-HDLc/HDLc ratio and 28-day mortality.

Models	Per-unit increase		Per-SD increase	
	OR (95%CI)	*P* value	OR (95%CI)	*P* value
**Model I**				
One line effect	1.04 (0.88, 1.23)	0.627	1.08 (0.78, 1.51)	0.627
**Model II**				
Turning point (K)	3.3		− 0.07	
non-HDLc/HDLc ratio < K	0.60 (0.37, 0.99)	0.043	0.36 (0.13, 0.97)	0.043
non-HDLc/HDLc ratio ≥ K	1.28 (1.01, 1.62)	0.039	1.64 (1.02, 2.63)	0.039
*P* value for LRT test*		0.023		0.023
95% CI for turning point	2.9, 3.9		− 0.26, 0.23	

Data were presented as OR (95% CI) *P* value; Model I, linear analysis; Model II, non-linear analysis. Adjusted for age (years), sex, weight, heart rate, lactate level, Apache IV score, SOFA score, septic shock and site of infection.

*CI* confidence interval, *OR* odds ratio, *LRT* logarithm likelihood ratio test.

*P < 0.05 indicates that model II is significantly different from Model I.

Using the generalized additive model, a U-shaped association between the non-HDLc/HDLc ratio and 28-day mortality was detected (Table 3). The linear regression model and a two-piece-wise linear regression model were compared, and the *P* value of the log-likelihood ratio test was 0.023. The 95% CI for turning point of the non-HDLc/HDLc ratio was 2.9–3.9 (Table 3). This result indicates that the two-piece-wise linear regression model should be used to fit the model.

The trend of the sensitivity analysis was consistent with that of the main analysis. In the supplementary analysis, we also analysed the 14-day mortality rate, and the results were generally similar to our main results (Fig. S1 and Table S1). Dummy variables were used to indicate missing covariate values. Similar results were obtained after considering the impact of missing data (Table S2). We generated an E-value to assess the sensitivity to unmeasured confounding. The primary findings were robust, unless there was an unmeasured confounder with an OR greater than 1.88.

## Discussion

This retrospective cohort study found a higher or lower non-HDLc/HDLc ratio was associated with a higher risk of 28-day mortality in patients with sepsis in the eICU-CRD database from 208 distinct ICUs across the United States between 2014 and 2015. The major finding was that the association between the non-HDLc/HDLc ratio and the risk of all-cause mortality was U-shaped, and the risk was highest in those with very low or very high non-HDLc/HDLc ratios. To our knowledge, this is the first study to report the association between the non-HDLc/HDLc ratio and 28-day mortality in septic patients.

Most studies investigating the relationship between levels of LDLc and the risk of death found no association^20–22^ or an inverse association^23–25^. A prospective population-based cohort study with 5 years of follow-up and a validation cohort of African American participants with a 4.25-year follow-up showed that neither HDLc nor LDLc was associated with mortality^20^. Data from 1948 to 1980 on 5209 men and women enrolled in the Framingham Heart Study found that the negative results for all-cause morality in the oldest age group appeared to be due to a negative relationship with LDLc levels rather than the protective effect of high HDLc levels^21^. You et al.^26^ included 356 patients with intracranial haemorrhage (mean follow-up = 0.22 years) and found that the LDLc/HDLc ratio was negatively correlated with all-cause mortality. Liu et al.^27^ recruited 3250 stroke patients and found a negative relationship between the LDLc/HDLc ratio and all-cause mortality.

Observational studies have established that HDLc is inversely associated with both cardiovascular disease and mortality across a wide range of concentrations^3^. A study by Bowe et al.^28^ included 1,764,986 men who were US veterans (mean follow-up = 9.1 years) and found a U-shaped relationship between HDL cholesterol and the risk of all-cause mortality. A study by Madsen et al.^29^ included 116,508 individuals from the general population. The association between HDL cholesterol and all-cause mortality was U-shaped, with both extremely high and low HDL cholesterol levels associated with high mortality. No previous study has examined HDLc or LDLc levels associated with the lowest risk of mortality in patients with sepsis. To our knowledge, although the relationship between blood lipid levels and mortality has been a concern, the association between the non-HDLc/HDLc ratio and 28-day mortality is still unclear, which prompted us to conduct the current study.

The 28-day mortality was 5.94% (43/724) in our cohort. Sepsis-related mortality was lower than that in other studies^30^, which may be related to severity of illness. A retrospective cohort study involving sepsis patients was conducted using the same database (eICU Collaborative Research Database)^31^. The in-hospital mortality was 16.7% (3828/22,868) in the total study cohort. The purpose of this research was to define persistent critical illness based on growth trajectories in patients with sepsis. Five latent classes were identified. Class 3 (51.7%), which had an in-hospital mortality rate of 6.7%, was characterized by moderate initial SOFA scores followed by decreasing severity of illness during the course of ICU stay. We have collected severity of illness indicators, including SOFA score, GCS score, Apache IV score and Acute Physiology Score III. Some important confounding factors can affect mortality in septic patients, including septic shock, lactate level, and comorbidities. In our study, those who lacked data on total cholesterol after ICU admission were excluded, so a large number of patients with sepsis were excluded.

We used non-HDL cholesterol rather than LDL cholesterol because our database measures TC and HDL cholesterol, which can be calculated by subtracting non-HDL cholesterol from it. Of the 724 patients included, 178 did not have LDL measurements. In addition, the most commonly used estimation method, the Friedewald equation, can be inaccurate^32^. Non-HDL and LDL cholesterol were correlated in studies with data on both variables (r = 0.93)^4^. In our study, the correlation coefficient between non-HDL and LDL cholesterol was 0.92 (95% CI 0.91–0.94), showing a high correlation. Non-HDL cholesterol predicts CHD risk at least as well as LDL cholesterol^33^ because it includes cholesterol in LDL, lipoprotein(a), intermediate-density lipoprotein, very-low-density lipoprotein and lipoprotein remnants and is thus a simple measure of cholesterol content within all atherogenic lipoproteins.

In addition, a recent study of 6941 hypertensive patients aged 65 years or older from a Chinese hypertension registry study who were not treated with lipid-lowering drugs showed that during a median follow-up of 1.72 years, 157 all-cause deaths occurred, and a U-shaped relationship between the LDLc/HDLc ratio and all-cause mortality was found^8^. This is similar to our result showing that the mortality rate associated with non-HDLc/HDLc ratios is not linear.

The association between low levels of non-HDLc and an increased risk of all-cause mortality could not be explained by causal effects. A possible explanation for our findings was that debilitation and illness caused a decrease in the levels of cholesterol^34,35^. Nonetheless, cholesterol-related risks are more complex and involve the interplay of several factors, such as cholesterol particle concentration, reverse cholesterol transport, and triglyceride-rich lipoproteins, to mention a few^36^. The observation that the association between non-HDLc/HDLc ratio and risk of death follows a U-shaped curve was not expected, and the mechanism underpinning the association of high HDLc and mortality is not clear. Our study did not examine the cause of death; however, we noted that the prevalence of comorbid diseases was not higher among those with a low non-HDLc/HDLc ratio, suggesting that comorbid disease events are unlikely to explain the increased risk of death in these subjects. We also noted a slightly higher percentage of patients with pulmonary infection and a higher average age among those with a low non-HDLc/HDLc ratio. Whether pulmonary infection or conditions associated with increased ageing (infections and chronic inflammation) explain the increased risk of death among patients with a low non-HDLc/HDLc ratio merits additional investigation.

The current retrospective cohort study is based on the eICU-CRD database and may be important for understanding non-HDLc/HDLc ratios in septic patients (i.e., when the focus is not limited to myocardial infarction or atherosclerotic cardiovascular disease).

### Study limitations

A common problem in observational studies is unmeasured confounders. As seen in Table 1, compared with subjects in the lowest tertile of the non-HDLc/HDLc ratio, subjects in the highest tertile were younger and had a higher admission weight and a higher rate of septic shock. These differences may be indicative of unmeasured confounders, such as income and medical insurance, which may affect the risk of 28-day mortality. Although ethnicity, in-hospital period, and age were included in the datasets, it is not possible to estimate the effect of unmeasured confounders on the ORs. We adjusted for possible confounding factors, including age (years), sex, weight, heart rate, lactate level, Apache IV score, SOFA score, septic shock and site of infection. We used E-value sensitivity analysis to quantify the potential implications of unmeasured confounders and found that an unmeasured confounder was unlikely to explain the entirety of the association. Additional limitations of our study include missing data for some variables. Nevertheless, we used contemporary methods to deal with missing data to minimize bias.

Another limitation is related to the fact that the diagnosis was based on the ICD-9 coding, which the responsible physician found relevant, and we did not have information concerning causes of death. Since we are examining mortality over a short period after the date of visit to the ICU, we did not find it beneficial to distinguish between cardiovascular and non-cardiovascular death.

Furthermore, lack of information on interventions during initial stabilization may have influenced non-HDLc/HDLc ratio levels and survival. It is noteworthy that the potential resulting from interventions would bias towards to the null and thus result in an underestimation of the association between non-HDLc/HDLc ratio level and mortality.

Finally, the 28-day mortality was 5.94% (43/724) in our cohort. The mortality of sepsis is lower than that in other studies. The possible reason is that the subjects included in this study excluded those who lacked total cholesterol after ICU admission, so that a large number of patients with sepsis were excluded. We acknowledge that the participants were patients referred to the emergency department for some reason, limiting the generalization of the findings to other populations.

## Conclusions

Data from the eICU-CRD database were used to identify 724 patients with sepsis. This study identified a nonlinear dose–response relationship between non-HDLc/HDLc ratios and 28-day mortality. The association between the non-HDLc/HDLc ratio and the 28-day mortality risk was a U-shaped curve. A higher or lower non-HDLc/HDLc ratio was associated with an increased risk of 28-day mortality.

## Supplementary Information

Supplementary Information.

## Data Availability

Data were fully available at https://eicu-crd.mit.edu/.
