# Supplementary material for: The association between the non-HDL-cholesterol to HDL-cholesterol ratio and 28-day mortality in sepsis patients: a cohort study
Source: Sci Rep. 2022 Mar 3;12:3476. doi: 10.1038/s41598-022-07459-y (PMC8894387; doi:10.1038/s41598-022-07459-y)
Supplement: Supplementary file 1 — Supplementary Information. [file 41598_2022_7459_MOESM1_ESM.docx]

**Title page**

**The association between the non-HDL-cholesterol to HDL-cholesterol ratio and 28-day mortality in sepsis patients: A cohort study**

Le Chang ^1^, Xinglin Chen^2, 3^ , Cheng Lian^4^
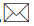


^1^Department of Orthopedics, Xijing Hospital, The Fourth Military Medical University, Changle West Road No. 127, Xi’an, 710032, Shaanxi, China; ^2^Department of Geriatrics, Union Hospital, Tongji Medical College, Huazhong University of Science and Technology, Wuhan, China; ^3^Department of Epidemiology and Biostatistics, Empower U, X&Y solutions Inc., Boston, USA; ^4^Department of Cardiology, Xi’an International Medical Center Hospital, Xitai Road No. 777, Xi’an, 710032, Shaanxi, China.

Corresponding author
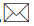


Cheng Lian

Department of Cardiology, Xi’an International Medical Center Hospital, Xitai Road No. 777, Xi’an, 710032, Shaanxi, China.

1. mail: cheng_lian2020@163.com

Supplementary material

**Supplemental material S1**


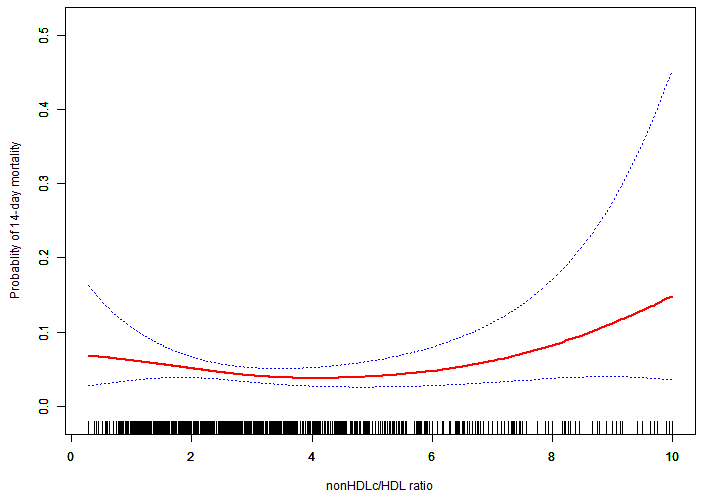


**Figure S1** Associations between the nonHDLc/HDLc ratio and 14-day mortality in all patients with sepsis. A threshold, nonlinear association between the nonHDLc/HDLc ratio and 14-day mortality was found in a generalized additive model (GAM). Solid rad line represents the smooth curve fit between variables. Blue bands represent the 95% of confidence interval from the fit. Adjusted for age (years), sex, weight, heart rate, lactate level, Apache IV score, SOFA score, septic shock and site of infection.

**Supplemental material S2**

**Table S1** Threshold effect analysis of the nonHDLc/HDLc ratio and 14-day mortality

| Models | Per-unit increase | | Per-SD increase | |
| --- | --- | --- | --- | --- |
|  | **OR (95%CI)** | ***P* value** | **OR (95%CI)** | ***P* value** |
| Model I | | | | |
| One line effect | 0.96 (0.80, 1.17) | 0.7093 | 0.93 (0.63, 1.37) | 0.7093 |
| Model II | | | | |
| Turning point (K) | 3.2 | | -0.12 | |
| NonHDLc/HDLc ratio < K | 0.46 (0.26, 0.81) | 0.0072 | 0.21 (0.07, 0.65) | 0.0072 |
| NonHDLc/HDLc ratio ≥ K | 1.28 (0.98, 1.67) | 0.0676 | 1.64 (0.96, 2.81) | 0.0676 |
| *P* value for LRT test* |  | 0.006 |  | 0.006 |
| 95% CI for turning point | 2.9, 3.7 | | -0.27, 0.15 | |

Data were presented as OR (95% CI) *P* value; Model I, linear analysis; Model II, non-linear analysis. Adjusted for age (years), sex, weight, heart rate, lactate level, Apache IV score, SOFA score, septic shock and site of infection. CI, confidence interval; OR, odds ratio; LRT, logarithm likelihood ratio test. * *P*<0.05 indicates that model II is significantly different from Model I.

**Supplemental material S3**

*Dealing with missing data*

**Table S2 Threshold effect analysis of the nonHDLc/HDLc ratio (per-unit increase) and 28-day mortality**

| Models | With missing data  (n=511) | | Dealing with missing data (n=713) | |
| --- | --- | --- | --- | --- |
|  | **OR (95%CI)** | ***P* value** | **OR (95%CI)** | ***P* value** |
| Model I | | | | |
| One line effect | 1.04 (0.87, 1.24) | 0.654 | 1.04 (0.88, 1.23) | 0.627 |
| Model II | | | | |
| Turning point (K) | 3.4 | | 3.3 | |
| NonHDLc/HDLc ratio < K | 0.53 (0.31, 0.88) | 0.013 | 0.60 (0.37, 0.99) | 0.043 |
| NonHDLc/HDLc ratio ≥ K | 1.40 (1.07, 1.82) | 0.013 | 1.28 (1.01, 1.62) | 0.039 |
| *P* value for LRT test* |  | 0.005 |  | 0.023 |
| 95% CI for turning point | 3.0, 4.1 | | 2.9, 3.9 | |

Data were presented as OR (95% CI) *P* value; Model I, linear analysis; Model II, non-linear analysis. Adjusted for age (years), sex, weight, heart rate, lactate level, Apache IV score, SOFA score, septic shock and site of infection. CI, confidence interval; OR, odds ratio; LRT, logarithm likelihood ratio test. * *P*<0.05 indicates that model II is significantly different from Model I. With missing data: The amount of missing values for the covariates were: 11 (1.52%) for admission weight, 5 (0.69%) for heart rate, 205 (28.3%) for lactate level and 7 (0.96%) for SOFA score. Dealing with missing data: Dummy variables were used to indicate missing covariate values, which was performed when continuous variables are missing more than 1%.

**Supplemental material S4**

**Table S3** Comparison of the severity of illness between included and excluded patients with sepsis

|  | **Total sepsis patients**  **(N=23136)** | **Excluded**  **(N=22412)** | **Included**  **(N=724)** | ***P* value** |
| --- | --- | --- | --- | --- |
| Apache IV score | 68.4 ± 26.4 | 68.2 ± 26.5 | 73.2 ± 23.9 | <0.001 |
| Acute Physiology Score III | 54.7 ± 24.9 | 54.5 ± 24.9 | 59.8 ± 23.2 | <0.001 |
| SOFA score | 2.0 (0.0-4.0) | 2.0 (0.0-4.0) | 3.0 (1.0-5.0) | 0.005 |
| GCS score | 12.6 ± 3.4 | 12.7 ± 3.4 | 11.9 ± 3.7 | <0.001 |
| Age (years) | 65.7 ± 16.2 | 65.7 ± 16.3 | 66.7 ± 15.1 | 0.085 |
| Length of ICU stay, days | 2.1 (1.1-4.1) | 2.1 (1.1-4.0) | 4.2 (2.9-7.8) | <0.001 |
| ICU Mortality |  |  |  | <0.001 |
| No | 20702 (89.5%) | 20022 (89.4%) | 680 (93.9%) |  |
| Yes | 2430 (10.5%) | 2386 (10.6%) | 44 (6.1%) |  |
| 28-day Mortality |  |  |  | <0.001 |
| No | 20732 (89.6%) | 20051 (89.5%) | 681 (94.1%) |  |
| Yes | 2404 (10.4%) | 2361 (10.5%) | 43 (5.9%) |  |

Data are expressed as the mean±SD, median (interquartile range), or percentage; GCS Glasgow Coma Scale; SOFA Sequential Organ Failure Assessment.
